# Research progress of electrode shapes in EWOD-based digital microfluidics

**DOI:** 10.1039/d3ra01817b

**Published:** 2023-06-05

**Authors:** Xingyue Wu, Dongbao Tang, Qianpei He, Luxuan Liu, Zhaoyuan Jia, Yuyu Tan

**Affiliations:** a School of Electrical Engineering, Ultra-fast/Micro-nano Technology and Advanced Laser Manufacturing Key Laboratory of Hunan Province, University of South China Hengyang 421001 China tanyuyu@usc.edu.cn; b Department of Comparative Medicine, School of Medicine, University of Washington Seattle WA USA

## Abstract

Digital microfluidics (DMF) is an innovative technology used for precise manipulation of liquid droplets. This technology has garnered significant attention in both industrial applications and scientific research due to its unique advantages. Among the key components of DMF, the driving electrode plays a role in facilitating droplet generation, transportation, splitting, merging, and mixing. This comprehensive review aims to present an in-depth understanding of the working principle of DMF particularly focusing on the Electrowetting On Dielectric (EWOD) method. Furthermore, it examines the impact of driving electrodes with varying geometries on droplet manipulation. By analyzing and comparing their characteristics, this review offers valuable insights and a fresh perspective on the design and application of driving electrodes in DMF based on the EWOD approach. Lastly, an assessment of the development trend and potential applications of DMF concludes the review, providing an outlook for future prospects in the field.

## Abstract 2

This review summarizes the working principles of DMF, and compares the effects of different electrode geometries on droplet manipulation.

## Graphical abstract

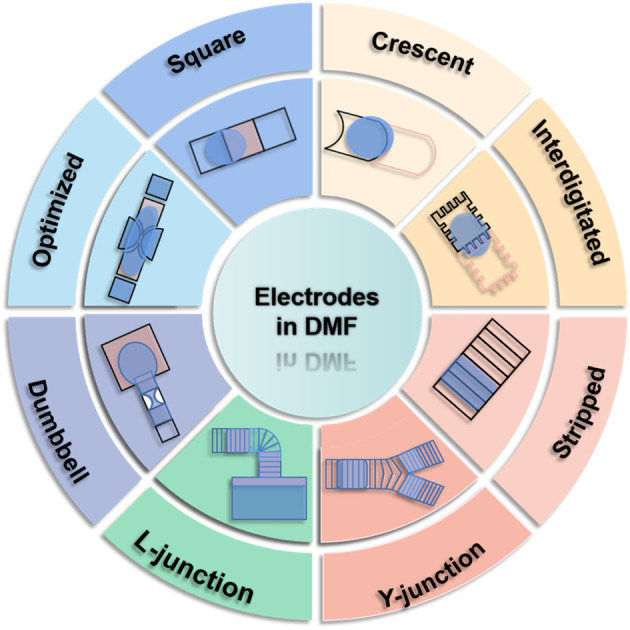

## Introduction

In recent years, microfluidic technology has emerged as a powerful tool for manipulating fluids at the micro-scale level. It offers significant advantages over conventional laboratory-based techniques, including enhanced efficiency in micro-operation, easy miniaturization and integration of analysis systems.^1–4^ As a subset of the microfluidic control system, Digital Microfluidics (DMF) simplifies the complex chip structure of traditional microfluidics and enables precise automated control of droplets, eliminating the need for pumps or microvalves. Utilisation of DMF allows for complex laboratory analysis by precisely controlling the transport the droplet in the microliter to nanoliter range,^5^ has received widespread attention due to its inherent advantages of simplified automation and seamless integration advantage,^6–8^ reduced sample and reagent consumption,^9,10^ minimised cross-contamination and high throughput capabilities.^11,12^ As a versatile platform, DMF is a dependable technological approach that offers immense potential and broad development opportunities in various fields, including nucleic acid analysis,^13,14^ proteomics,^15,16^ medical diagnosis,^17–19^ biological analysis,^20^ and point-of-care testing.^21^

DMF exhibits two distinct configurations, the closed type and the open type.^22,23^ In the closed type configuration, the droplet is sandwiched between the ground electrode of the top plate and the driving electrode of the bottom plate. This design enables four essential droplet operations: dispensing, transport, splitting, and merging, making it suitable for closed DMF devices. Additionally, the cutting of droplet is influenced by the height between the two plates.^24^ On the other hand, the open type configuration features the ground and the driving electrodes situated on a single bottom plate. However, the open type configuration typically faces limitations in splitting and dispensing due to a lack of sufficient shear forces. Moreover, the droplets in the open type are more prone to evaporation. Nevertheless, the open type offers advantages such as direct addition of droplets to the target position instead of moving to the target position. This feature facilitates rapid mixing of samples and reagents and provides easier connectivity with other instruments.^25^

In the closed type configuration of a DMF chip, the bottom plate is typically consists of four essential components in both configurations: substrate, electrode, dielectric layer, and hydrophobic layer. The dielectric layer is strategically positioned above the driving electrodes to prevent undesired electrolysis effects on the droplet. Additionally, a hydrophobic coating is applied to the dielectric layer, which effectively reduces the adhesion of the droplets and thus enhances the overall transport speed. This hydrophobic coating promotes efficient droplet movement within the closed DMF system.

In the fabrication process of DMF chips, the choice of substrate material plays a crucial role in determining the chip's manufacturing process and electrode array design. Glass is a commonly preferred substrate material for DMF chips due to its chemical inertness,^26^ while its transparency facilitates optical detection. Alternatively, printed circuit boards have emerged as a viable option for DMF chip substrates primarily due to their affordability, ease of integration with other electrical interfaces, and simplified wiring characteristics.^27–30^ Furthermore, flexible materials and paper have also been used as substrates for DMF chips.^31,32^ These materials enable droplet manipulation on non-planar surfaces, expanding the possibilities for droplet control and reducing costs. Selection of appropriate dielectric layer materials is essential for achieving optimal operational performance and functional expansion of the system. One such material is off-stoichiometric thiol–ene (OSTE) polymer, which offers adjustable surface and volume characteristics. This property enables direct combination of microchannels, expanding the practical connection between DMF and other microchannels.^33^ Additionally, food packaging films have emerged as cost-effective alternatives for dielectric layers, significantly reducing the overall expense of the DMF chip fabrication process.^34^

The driving electrode is a pivotal component of the DMF chip as it enables the crucial droplet manipulation functionality. The effective operation of the DMF chip relies on the precise control of droplets on the electrodes. Typically, the electrodes on the chip are fabricated using metal etching, which offers high precision and the ability to create intricate patterns. However, this method can be costly. In general, the driving electrode array of DMF is divided into two categories: the reservoir and the reaction electrodes. The reservoir electrodes have a larger surface area making it convenient to store reaction reagents or collect waste liquid. Through careful control of electrode switching, the DMF chip facilitates the desired movement and manipulation of droplets, enabling various functionalities and applications.

Furthermore, the size of the generated droplet is closely related to the dimensions of the electrode. The shape and quantity of control electrodes can also affect the droplet volume. The design of reaction electrodes will usually determine their layout or number according to the specific experimental requirements. The more complex the required function, the higher the requirements for droplet control, and the more requirements on electrodes, such as size and the number of electrodes. For example, the smaller the spacing between the top and bottom plates and the larger the electrode size, the better the droplet splitting effect.

The conventional chip adopts a discrete electrode structure. Each electrode is independently controlled. Although the operation is simple and convenient, the control circuit of the electrode is arranged very complexly when the number of electrodes is too large. For the portable DMF platform, it may cause large volume, and the operation of multiple droplets is troublesome. Some researchers have used large-scale electrode arrays to improve the chip's ability to control droplets in parallel,^35,36^ which helps to synchronize the experiment and shorten the experimental period. It is an essential direction for the development of DMF,^37^ and facilitates high-throughput analysis on the DMF chip.

The driving electrode in DMF directly affects the droplet manipulations. Meanwhile, the different shapes or sizes of the electrodes show different properties in the experimental process, so the design and preparation of suitable driving electrodes are crucial. This paper reviews the advances in driving electrodes in the DMF based on the EWOD in recent years.

## Principles of DMF

In 1993, to eliminate the occurrence of electrolysis in electrowetting, Berge added a dielectric layer to the electrowetting model,^38^ which changed the wetting characteristics of the droplets on the dielectric layer. Those EWOD devices have been widely used in DMF,^39^ optics,^40,41^ display.^42,43^

DMF is a droplet control technique for controlling discrete droplets through droplet production, delivery, division, merging, and other functions.^24,44–48^ According to different droplet control principles, the methods of manipulating droplets can be divided into EWOD,^49,50^ surfaces acoustic wave,^51^ thermocapillary forces transmission,^52^ dielectrophoresis,^53,54^ magnetic force.^55^ EWOD has the advantages of no heat, fast switching response, no mechanical parts, and low power consumption. It is thus considered a practical DMF driving method. The related principles of DMF based on EWOD are introduced.

**Fig. 1:**
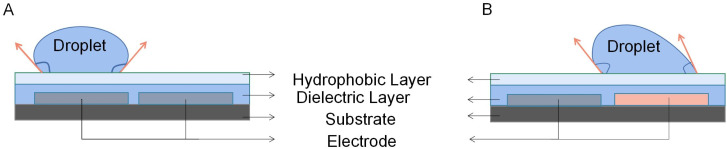
Principle of droplet control in DMF based on EWOD. (A) The state of the droplet when electrodes do not apply voltage. (B) The state of the droplet when electrodes are applied with a voltage.

The DMF technique based on EWOD was first proposed by Pollack^56^ and Lee.^57^ The basic principle is shown in Fig. 1. When applying a voltage to the driving electrode under the dielectric layer, the voltage changes the surface free energy of the solid–liquid interface and reduces surface tension on the droplet, it changes the contact angle so that the droplet moves in the direction of the driving electrode.^58^

The Lippmann–Young Equation describes the relationship between the contact angle and the voltage:^59^1
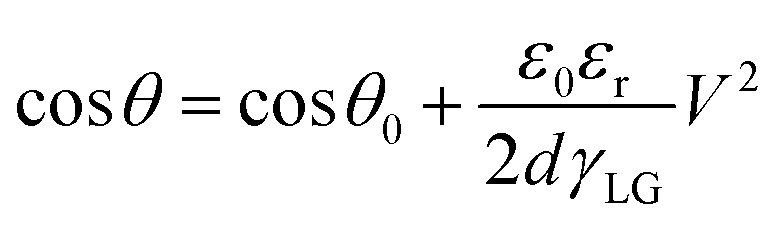


*θ* and *θ*_0_ represents the contact angle size of the applied drive voltage and the unapplied voltage, *γ*_LG_ is the interfacial tension coefficient of the liquid and gas, *ε*_r_ is the relative dielectric constant of the dielectric layer and *ε*_0_ is the vacuum dielectric constant, *d* represents the dielectric layer thickness, and *V* indicates the voltage across the dielectric layer.

The electric wetting force of the droplet in the forward direction when voltage is applied:^60,61^2
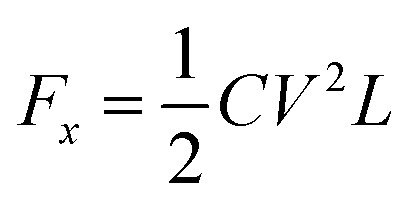


*C* indicates the unit equivalent capacitance of the dielectric layer, *L* is the string length of the three-phase contact line where the droplet cross-section circle is located on the conductive drive electrode.

Jones proposed aninterpretation of electrowetting that allows a better understanding of the physics of droplet actuation.^62,63^ He proposed that the electrostatic force generated by charge accumulation at the three-phase contact line causes a change in the tension, which causes a change in the contact angle.

The electrostatic force can be calculated by integrating the Maxwell-stress tensor *T*_ik_:

3 *T*_ik_ = *ε*(*E*_i_*E*_k_ − 0.5*δ*_ik_*E*^2^)

*δ*_ik_ is the Kronecker delta function, i and k refer to pairs of *x*, *y* and *z* axes, *E* is the electric field surrounding the droplet.

Another explanation is the Energy minimization method: the sum of surface and electric field energy is minimized by a variational principle. Eqn (1) is also obtained, which is mainly suitable for analyzing the steady-state process of the whole droplet.

When the electrode is turned on, it is known from eqn (1) that the electrowetting of droplets changes, and the contact angles differ on the left and right sides. The pressure difference generated inside drives the droplet along the set path.

Numerous papers about DMF have been published, but some problems still need to be solved.^64^ For example, high drive voltage may cause electrode breakdown, and the volume deviation of the droplet dispensing on the square electrode is ∼30%, which cannot meet the experimental requirements of high precision. Droplet splitting is unequal in volume, and the inconsistency is ∼10%, not enough to achieve the 5% requirement in the usual experiments.

With the improvement of actual research needs, it is vital to meet the requirements of high-throughput and precision droplet control. Different geometries of driving electrodes provide an idea to solve the above problems.

### Effect of different geometries of electrodes on droplet driving

With the accelerated development of DMF and the increasing application fields, many different structural and functional requirements are emerging. Electrode shape and size directly determine the electric field distribution and parameters of droplet motion. Changing the geometry of the driving electrode is a means to regulate the performance of droplet manipulation. Because of the limitations of driving electrodes, electrodes with different geometries have been designed to meet additional experimental requirements.

#### Square electrode

**Fig. 2:**
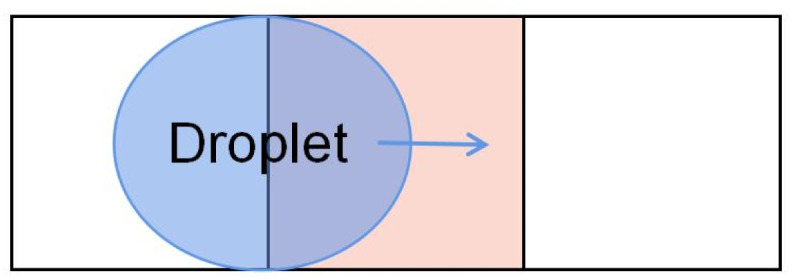
Droplet was moved over square electrodes.

The square electrode is symmetrical with a simple shape and is convenient to arrange the electrode array. The electrode preparation is easier to achieve in the case of low accuracies, such as printed circuit board, because it is no complicated line design. The square electrode is widely used in DMF,^65–67^ and the general process is shown in Fig. 2.

DMF devices using square electrodes are only suitable for droplets of one size. Furthermore, the electric field of the square electrode has a relatively gentle potential energy gradient change and a slow liquid transport rate, and the droplet may not move at the gap.^68^ Thus, droplet transport's continuity and velocity stability must be improved. In response to the problems of square electrodes, researchers tried to propose some electrodes that can operate droplets more quickly, stably, and accurately.

#### Interdigitated electrode

**Fig. 3:**
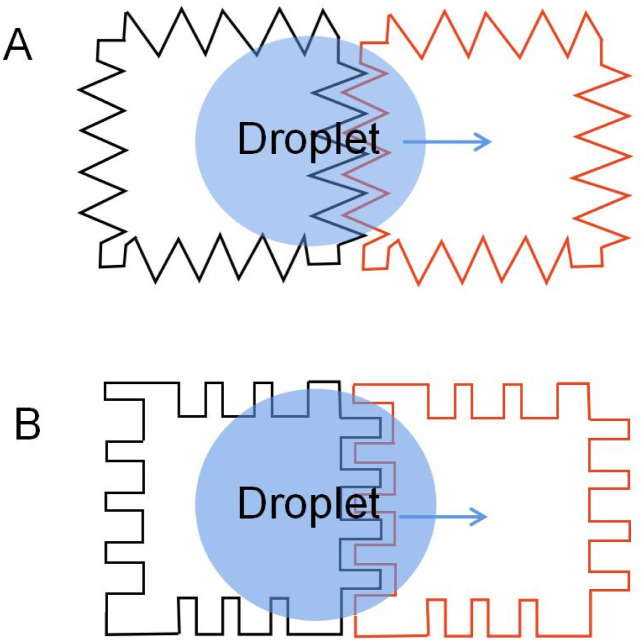
Two common interdigitated electrodes: (A) electrodes with jagged edges. (B) Electrodes with interdigitated fingers.

In 2000, Pollack *et al.* designed a DMF device with an array of jagged control electrodes as the driving electrode,^56^ such electrodes with jagged edges or interdigitated fingers were accomplished, and the electrode properties were also investigated. Common interdigitated electrodes are shown in Fig. 3.

To complete a continuous movement of the droplet in the DMF device, the droplet edge must be able to contact the next energized electrode. Sometimes the droplet moves slowly and is not touched when the next electrode is energized, which causes the droplet to stop moving. Moon used an interdigitated electrode to solve this problem.^69^ Interdigitated electrode is designed to allow the droplet's edge to come in contact with the next electrode when the droplet rests in the middle of the two electrodes. When energized, the interdigitated electrodes broke the droplet equilibrium, achieving the droplet's continuous movement.

Jang examined the electrode geometry's effect on the EWOD device's droplet velocity.^70^ Compared to the contact line lengths of the different-shaped electrodes, it was found that the contact line of the interdigitated electrodes was longer than the square electrodes. According to eqn (2), the force on the droplet is related to the length of the contact line. The longer the contact line length, the greater the force on the droplet. Then the velocity of the droplets on the different electrode arrays was tested: a linear array of square electrodes with dimensions of 1 × 1 mm, and an array of interdigitated electrodes having either two or three fingers measuring 0.2 mm and 0.07 mm in length and width. It was demonstrated that the droplet speed of the interdigitated electrode could reach 8.17 mm s^−1^, while the square electrode is 7.25 mm s^−1^, and droplet velocity can be improved by using an interdigitated electrode. The experimental results were consistent with the theory.

**Fig. 4:**
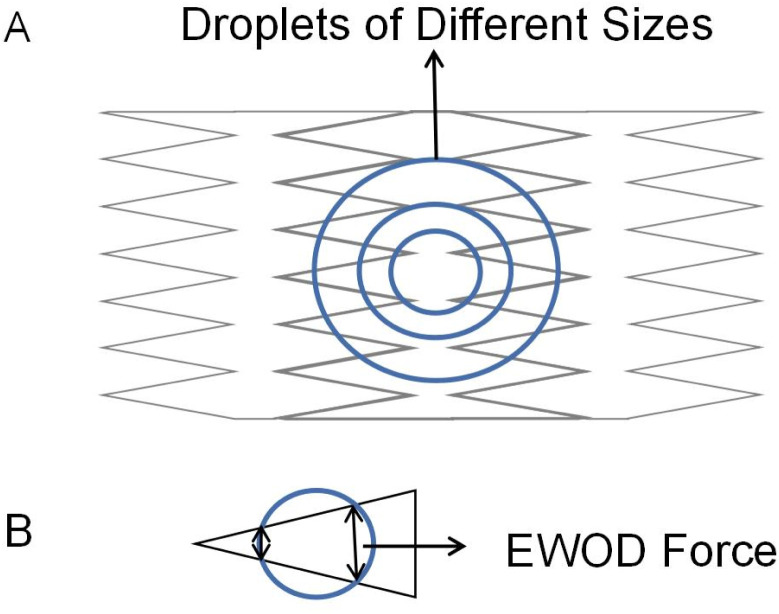
(A) Triangle interdigitated electrodes for size-variable droplet actuation. (B) The droplet is actuated by triangle fingers electrode.

In addition to increasing the transport velocity of droplets on the DMF platform, interdigitated electrodes also improve DMF's flexibility to manipulate different droplets. Chen attempts to control droplets of various sizes in the same electrode array with a rectangular interdigitated electrode.^71^ Verified that rectangular interdigitated electrodes with finger length widths of 0.6 and 0.2 mm could achieve movement of 27 nL to 190 nL droplets. Triangle interdigitated electrodes based on the rectangular interdigitated electrode are designed to drive smaller droplets, as shown in Fig. 4A. The size of the electrode determines the minimum liquid volume that can be driven. The tip of the triangle is small. The EWOD force is not balanced because of the geometry so that it can drive smaller droplets (Fig. 4B). It was found that triangle interdigitated electrodes can actuate droplets of 15 nL, as small as 1/36 of that actuated by the square electrode. It partially solves the problem of flexibility of DMF and droplets of only one size.

Based on the contact line elasticity theory, Berthier derived the geometric conditions for the dent size of the interdigitated electrodes in the EWOD microsystem.^72^ This condition suggests that the nondimensional dimension ratio of dimple length to width should be large enough. It verifies that different interdigitated electrodes found in the literature all met this standard.

**Fig. 5:**
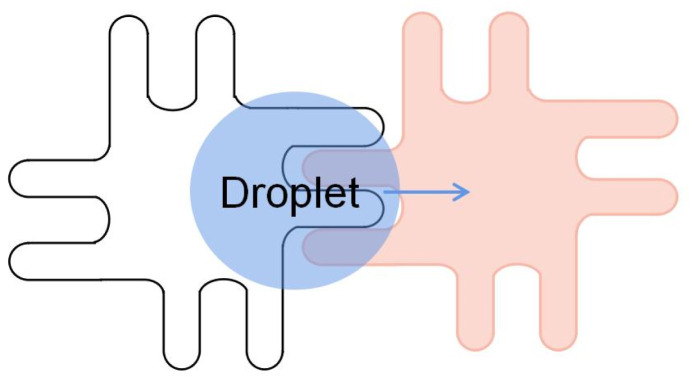
Symmetric interlocking fingers electrode.

Lin proposed an electrode with symmetric interlocking fingers, which were 35 μm long and 25 μm wide,^73^ as shown in Fig. 5. The symmetric shape of the electrode allows for a more flexible electrode layout. A smaller layout of densely interlocked electrodes can be installed in the chip, enabling the transfer of smaller droplet volumes and allowing the DMF chip to drive the microliter droplets better. Dispensing 300 pL droplets from reservoirs was accomplished with as little as 11.4 V. The actuation threshold voltage was 7.2 V. A symmetric interlocking finger electrode design allows more interleaving and less-driving threshold voltage between adjacent electrodes.

Kremers designed a portable DMF platform named PortaDrop, the electrodes on the platform are gold interdigitated electrodes with a width and gap design of 5 μm.^74^ Droplet dispensing and transport on the platform were examined. At the 200 V voltage, the mean value of the droplet volume was 249.5 nL with a standard deviation of 20.8 nL, and the error was 8.3%. The maximum average droplet velocity was 32.9 mm s^−1^.

Compared with square electrodes, the interdigitated electrode array can increase the overlap between the electrodes, that is, the contact lines of the droplets. According to formula (2), the value of the effective three-phase contact line L becomes larger, so the electrowetting force becomes stronger, thus giving the droplet a faster velocity. Moreover, the electrode plane extends to the next electrode, avoiding the droplet stop due to the hydrophobic gap between the electrodes, making the droplet transport more continuous. The droplets on interdigitated electrodes run faster and have a more extensive volume range. However, interdigitated electrodes have clear electrode outlines and are sometimes easily broken down by voltage.

#### Crescent electrode

**Fig. 6:**
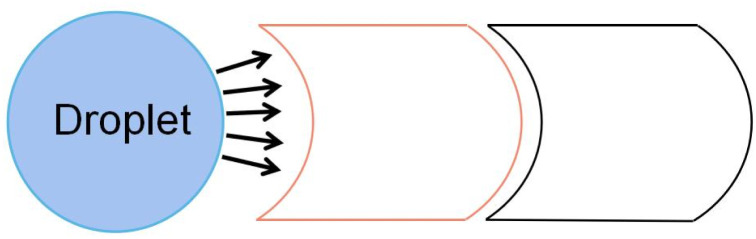
Driving force distribution of droplets on crescent electrodes.

In 2010, Rajabi designed a new crescent-shaped electrode by combining numerical simulations and experiments with studying the effect of electrode shape in the system.^75^ As shown in Fig. 6, because the circular arc of the crescent electrode is similar to the profile of the droplet, the new electrode can increase the overlap between the adjacent electrodes. Increasing the length of the three-phase contact line provides a more oversized uniform driving force on the contact line, so the crescent electrode drives at a higher speed at the same voltage. Uniform driving force reduces the stretching of droplets during transport, increasing the droplet's transporting speed. The droplet velocity was increased up to twice compared to the square electrode.

Due to the crescent electrodes' excellent performance, researchers have explored many crescent electrodes. Xu investigated the droplet movement on the crescent electrodes, which experimentally showed faster droplet movement under the same driving voltage.^76^ Among the three drive electrodes: square, interdigitated, and crescent electrode, the crescent electrode can provide a higher droplet velocity at a relatively small drive voltage and the highest continuity. At the 40 V voltage, the droplet transport speed on the crescent electrode is about ∼120 mm s^−1^, compared to ∼20 mm s^−1^ on the square electrode and ∼50 mm s^−1^ on the interdigitated electrode. In 100 attempts, the crescent electrode can achieve 95 times continuous movement without repeat actuation of the drive electrodes. The number of successful movements is higher than the other electrode. The superior performance of the crescent electrode on the droplet drive was demonstrated.

On the droplet splitting, the crescent electrode performed even better.^77^ At a channel height of 50 μm, the crescent electrode can split droplets successfully seven times in ten times, compared to only four times for the square electrode. The square electrode's minimum split voltage with a droplet volume of 1.8 μL was 72 V. In contrast, the crescent electrode was 62 V. It partly addresses the problem of low droplet splitting success rate and high splitting voltage in the square electrode.

**Fig. 7:**
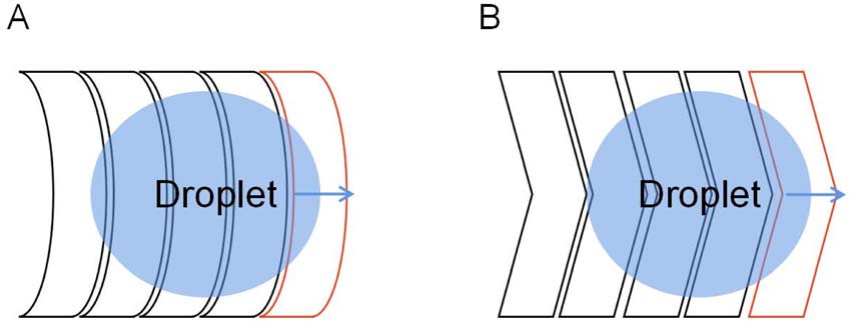
Two patterns of crescent electrodes. (A) Arc design. (B) Chevron design.

The development of large-scale DMF devices requires many electrodes, and the increase of electrodes requires more circuits to control the electrodes, leading to a larger volume of DMF. To reduce the complex control circuit design of the electrodes, Böhringer designed, fabricated and characterized an open AC EWOD platform with two kinds of crescent electrodes.^78^ As shown in Fig. 7, only two electrodes are required to transport the droplet using anisotropic electrode patterning. When applied sinusoidal AC voltage, the droplets can be continuously pumped to the moved without controlling circuits. Droplet transport, frequency response, and transport ability on inclined surfaces were tested. At 20 Hz external AC frequency, the droplet transport speed of 15 μL can reach 20 mm s^−1^. In the inclined plane of 90°, the tiny droplet (∼5 μL) can move laterally on the surface without sliding.

**Fig. 8:**
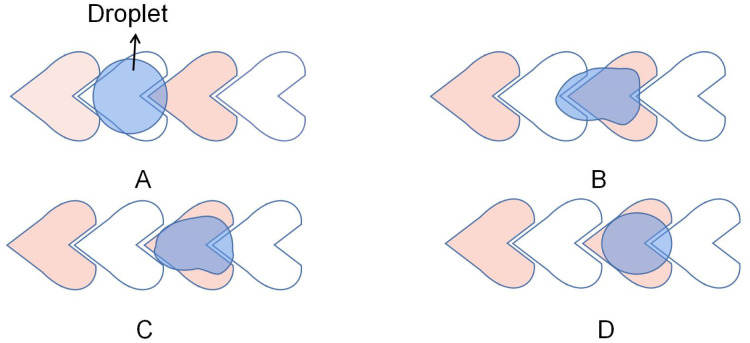
The transport of droplets on heart-shaped electrodes.

Similarly, Wang *et al.* designed a heart-shaped electrode for the unidirectional droplet movement to simplify the circuit.^79^ As shown in Fig. 8, similar to the crescent electrode, which is the crescent electrode with a larger arc, the geometry of the electrode ensures that the droplets coincide more with the front electrode than the rear electrode, so a forward driving force can be obtained. The velocity of the 340 nL droplet was ∼20 mm s^−1^ at a voltage of 90 V. This design utilizes alternating interconnection of electrodes in the electrode array and alternating drive signals, simplifying the circuit wiring. This electrode design provides a simple method for large-scale DMF integration.

**Fig. 9:**
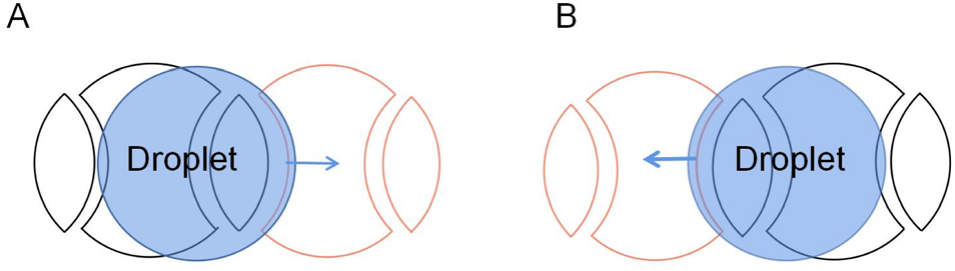
Droplet movement on the improved crescent electrode in two directions. (A) The droplet moves forward. (B) The droplet moves backward.

There are also deficiencies in the droplet transport of the crescent electrodes. The driving force provided by the crescent electrode is unidirectional, while the droplets in the actual transport process often do not move in one direction. For the crescent electrode that is only suitable for single-directional operations, they proposed an improved crescent electrode consisting of two different shape electrodes to solve the problem.^80^ As shown in Fig. 9, the symmetrical electrode shape makes the droplet submissive in both directions of motion on the electrode, so the droplet's bidirectional motion has the same driving performance.

**Fig. 10:**
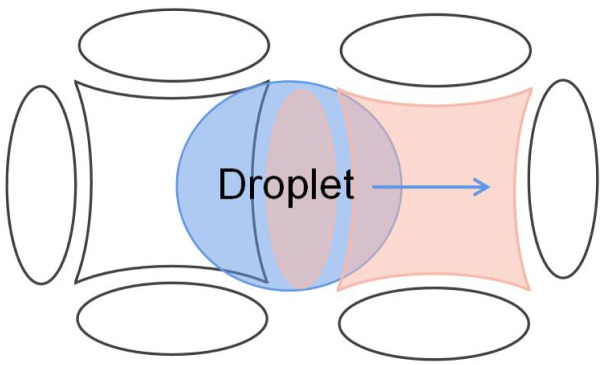
Combined electrode pattern.

Because of the electrode geometry, the improved crescent electrode has an inconvenient layout and can not transport droplets in other directions except for moving back and forth. To realize the free switch of the droplet operation direction in the electrode array, which allows the droplet to move in multiple directions, Wang *et al.* proposed and designed a combined electrode to improve the transport efficiency of the droplets.^81^ As shown in Fig. 10, based on the improved crescent electrode, the combined electrode adds elliptical electrodes above and below the intermediate electrode, which consists of a curved intermediate electrode and four elliptical electrodes. By applying a sequential drive voltage in the curved quadrilateral combined electrode array, the free control of the droplets in the up and down and left and right directions can be realized. Experiments show that when the DC voltage is 194 V, the average velocity of the propylene carbonate droplets in air is 25 μm s^−1^. When the average velocity in oil is 260 μm s^−1^, the droplet velocity can be effectively increased.

The combined electrode is graphically symmetrical, and the arrangement in the electrode array is more advantageous due to the symmetrical shape of the combined electrodes.

#### Stripped electrode

In 2016, Nahar *et al.* proposed a stripped electrode that drives the droplets and boosts the transport speed of the droplets by actuating different numbers of stripped electrodes.^82^ The distance of the droplet moves is only the width of the electrode. Because the striped electrode's width is smaller, the transport distance on the stripped electrode is less than the square electrode. The smaller distance limits the deformation of the droplet and does not break during transport because of the deformation. Thus it is more stable during droplet transport on the stripped electrode.^83^ Experiments show that using two or five stripped electrodes can reach 40 mm s^−1^ at the average speed of the square electrode is 20 mm s^−1^, which significantly improves the transport speed of the droplets, demonstrating the superiority of stripped electrodes in droplet transport.

**Fig. 11:**
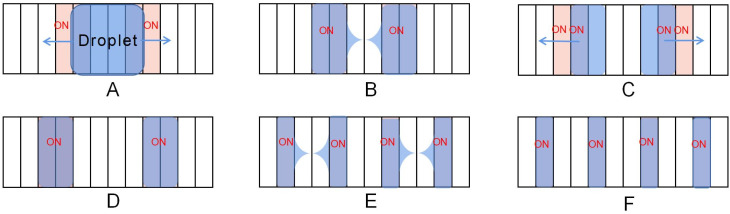
The generation process of droplets on the stripped electrode: (A–C) the parent droplet is divided into two droplets. (D–F) The droplet is divided into four sub-droplets of comparable size to the stripped electrode.

More studies were conducted on the design of this electrode. Stripped electrodes can be used for droplet generation. Guan proposed a DMF system consisting of stripped electrodes for droplet generation.^84^ The design consisted of 12 stripped electrodes of equal size. The entire droplet generation includes two separations and one delivery, as shown in Fig. 11. First, the parent droplet was placed in the center of the device with a size equal to the four stripped electrodes. In the end, two separations were performed, yielding four sub-droplets of comparable size to a single stripped electrode. The sub-droplet size deviation from that of the stripped electrode is smaller than 3.5%, showing that this electrode can be used for parallel droplet production due to its excellent precision and control ability.

This stripped electrode is designed to improve droplet transmission efficiency. It is simple to fabricate and performs well in splitting and generating droplets. However, in many cases, one droplet can cover 2–4 driving electrodes simultaneously because of the small width of the stripped electrodes, which increases the difficulty of the droplet manipulation.

#### Virtual electrode

Chiou *et al.* proposed the optoelectrowetting (OEW) device.^85^ OEW changes the conductivity of the photoconductive material land and reduce the impedance through the beam irradiation so that the electrode can be energized through a light projection pattern. On this device, a microliter of size droplet was successfully transported. In 2008, nanoliter-of-size droplet transport, separation, and multi-droplet operation under optical control were achieved.^86^ In 2015, Pei *et al.* designed an OEW device with optimal photoconductor thickness using a distributed circuit mode,^87^ which reduced optical power by 200 times and allowed commercial projectors to create virtual system electrodes for large-scale, parallel droplet operation. Virtual electrodes are also demonstrated for the parallel transport of droplets moving greater than 1 cm s^−1^ and the experimental process of detecting the herpes simplex virus using an isothermal polymerase chain reaction.

The optical image is projected in the desired shape to the surface of the light-actuated DMF device, locally changing the surface wetting through the electrowetting mechanism and controlling the droplets with virtual electrodes, which is helpful for parallel processes of a large-scale droplet operation. Virtual electrodes do not have a specific electrode mode. It can change the optical mode according to the requirements to change the shape of the electrode and achieve different operational requirements. Contrasted with the current droplet operation methods, the virtual electrode technology has the advantage of being easy to manufacture, simple consumable materials and real-time and reconfigurable properties. However, the increased temperature of the droplet due to the absorption heating of the photosensitive layer may affect many temperature-sensitive chemical or biological reactions.

#### TCC reservoir, L-junction and Y-junction electrode

Nikapitiya investigated how to improve the performance of droplet dispensing and splitting,^88,89^ and derived two conditions: (1) the volume attached to the liquid should be minimal when splitting; (2) the neck contraction at division is always in the same position. Secondly, those electrodes were designed and tested according to the proposed conditions.

**Fig. 12:**
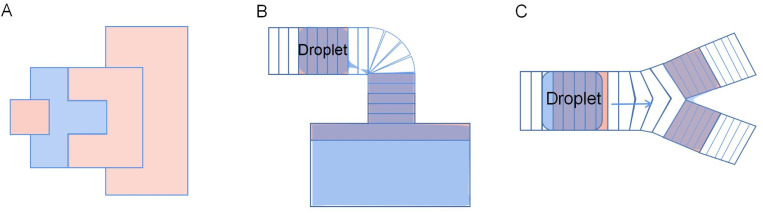
The diagrams of Electrodes: (A) TCC reservoir electrode. (B) L-junction electrodes. (C) Y-junction electrodes.

A novel reservoir electrode was designed to improve the volume and consistency of droplet dispensing. As shown in Fig. 12A, the reservoir electrode comprises one T-shape electrode and two C-shape electrodes and is arranged neatly, so it is named a TCC reservoir. A T-shape electrode serves as a cut point, and two C-shape electrodes serve as a reservoir. Half the square electrode was placed inside the T-shape electrode to reduce the cutting length and liquid trailing volume. During the droplet dispensing process, the square and C-shape electrodes are opened to form an extrusion on the droplet. And the unique and symmetrical electrode shape makes the cutting length of the droplet on the T-shape electrode shorter and smaller, ensuring that the droplet breaks in a fixed position on the T-shape electrode to make the consistent volume of each droplet dispensing. Test the average volume error of 50 droplets from the reservoir. The error of TCC reservoir design is 0.083%, and higher dispensing droplet accuracy than 8.3% of interdigitated electrodes, while the conventional design error is 34%. It dramatically improves the volume accuracy of the dispensing droplets, showing excellent consistency. Furthermore, the droplets are closer to the size of the generating electrode because of the much shorter cutting length formed.

Guan conducted numerical analysis and experimental comparison of droplet dispensing of the conventional, stripped electrode, and TCC reservoir, then studied the accuracy of the droplet.^90^ In the experiment, the traditional reservoir can also achieve good volume consistency. However, the final droplet volume is 30% larger than the target volume due to the formation of a longer neck when the droplet is generated. The inconsistency of the stripped electrode is 0.437%, which is better than that of the conventional reservoir, but it also has the problem of large droplet volume. The TCC reservoir design was the best of the three because its volume showed excellent consistency of 0.333%, and the droplet volume was less than 5% larger than the target volume due to the shorter neck formed during the cutting. Fully demonstrate the superior electrode design for droplet dispensing in droplet volume accuracy, which is particularly beneficial for applications where both volume consistency and accuracy are crucial.

To reduce droplet dispensing time, an L-junction electrode is developed. As shown in Fig. 12B, the L-junction electrode is composed of stripped and fan-shaped electrodes and which are arranged as L-shaped. They fix the fracture position using the fan-shaped electrodes and provide rapid droplet transport using stripped electrodes that can provide high-throughput droplet production. The L-junction device creates 90 droplets per second with a 0.81% error in volume, while the TCC reservoir creates three droplets per second. The speed has been increased by 30 times. Under the premise of ensuring volume accuracy, the electrode design dramatically shortens the time of droplet dispensing.

The Y-junction electrode is arranged by stripped electrodes of different sizes into Y-shaped. Compared to the L-shaped electrode, its shape does not change much, but changes its arrangement and combination. During the droplet splitting, the droplet moves forward onto two electrified electrode arrays and achieves droplet splitting due to the angle between the two electrode channels. As shown in Fig. 12C, the left and right branches' corresponding electrodes are connected in a slightly larger electrode array. During the splitting, the droplet divides rapidly due to the droplet being pulled into two branches. Conventional splitting by two square electrodes takes 125 ms, but splitting at Y-junction takes only 5 ms, increasing the speed of the droplet splitting significantly.

TCC reservoir, L-junction, and Y-junction electrodes can achieve droplet generation and split faster and more precisely than square electrodes. It is difficult for traditional electrodes to control the droplet size when the droplets are generated or split, which affects the application of DMF chips in the field of micro refinement. In all three electrode designs, the new electrode shape is used to ensure disconnection at a fixed location and increase liquid velocity, thereby accelerating the process of droplet manipulation. TCC reservoir, L-junction, and Y-junction electrodes provide new choices for producing precise volume droplets and high-throughput droplet applications in DMF.

#### Dumbbell electrode

**Fig. 13:**
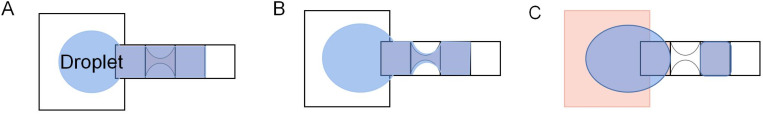
The generation process of the droplet in the dumbbell electrode. (A) The liquid extruded from the reservoir along the actuated sequence of electrodes. (B) Connect the reservoir and the electrode producing the droplet to form a neck on the center dumbbell electrode. (C) Switch off the dumbbell electrode, and then the liquid breaks by traction on both sides to form a droplet.

Wang *et al.* designed a dumbbell electrode to improve the uniformity of the droplet volume.^91^ The dispensing process of the droplet is shown in Fig. 13. He proposed that the performance improvement in droplet dispensing and splitting is mainly due to the fixed position of the pinch-off point, shortened liquid tail length, and the uniform and stable shape of the droplet neck. Furthermore, a novel electrode has been developed to control droplet contraction by the shape and position of the pinch-off point. The liquid connected the reservoir and the site where the droplet was produced, forming an elongated and stable neck, then turned the central dumbbell electrode hydrophobic. The cutting electrodes made the liquid break off at the thinnest position of the central dumbbell to obtain a droplet at the creation site. The droplet dispensing process of the electrode takes one additional step, which is to fix the neck during the division of the droplet. This step ensures that the breaking point of the droplet is always on the neck electrode and makes the droplet more stable during the division process. In addition, the less liquid on the neck electrode reduces the volume difference produced by the droplet's tail.

**Fig. 14:**
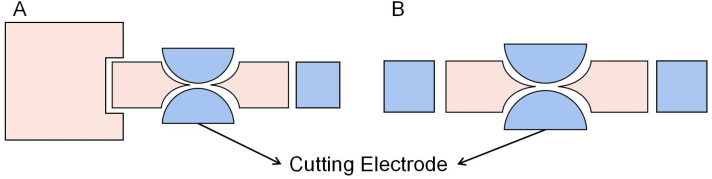
(A) The optimized electrode was used for the droplet dispensing. (B) The optimized electrode was used for the droplet splitting.

Using the same electrode design, Wang *et al.* optimized the electrode geometry to achieve a more accurate droplet dispensing and splitting volume.^92^ As shown in Fig. 14, the optimized electrode designed the neck of the central dumbbell electrode to be thinner until the electrode was broken and divided into two electrodes. Then uses the cutting electrode of different radius to fix the neck to optimize the droplet distribution and splitting. On dispensing, the contracted fluid coincides with the shape of the cutting electrode, and the liquid column is disconnected at the fixed point. The average droplet volume assigned by square electrodes is approximately 27% larger than the target volume, with an error of 9%. After optimization, dispensing inaccuracy was reduced by at least ten times, the inaccuracy minimum to 1.33%, inconsistency decreased by at least five times, and the minimum to 0.532%. The volume consisting of the two sub-droplets was tested in the splitting. The volume inconsistency decreased from 13.3% to 1.8%, with the best results of 1.12%.

The optimized electrodes improve the accuracy of droplet volume compared to square electrodes. Moreover, compared with the Y-junction electrode, the optimized electrode solved the shortcoming that splitting can only be done in a single direction. However, the complex design of splitting electrodes may not be convenient enough.

The TCC reservoir, L-junction and Y-junction electrodes, dumbbell electrodes, and optimized electrodes are all designed to dispense and split droplets. Electrodes of different shapes can ensure the smooth breakup of the droplets and control the pinching-off point of droplets to remain unchanged, thereby producing higher volumetric accuracy and meeting the experimental requirements of high-volume accuracy. However, there may also be the disadvantage of an inconvenient layout on the chip due to the particular shape of the electrodes.

### Characteristic contrast of driving electrodes

In order to better control the droplets and meet different experimental requirements, DMF electrodes of different shapes were designed to be applied to manipulate the droplets. The characteristics of the various electrodes for droplet driving in DMF are summarized in Table 1.

**Table 1:** Comparison of the properties of the electrodes with different geometries

Shape	Operation	Electrode properties	Ref.
Square electrode	Transporting	• Favorable for the layout of the electrodes	60
	Dispensing	• Convenient to manufacture	84
	Splitting		89
Interdigitated electrode	Transporting	• Increase the overlap between the electrodes	74
	Dispensing	• Suitable for droplets of different sizes	
Crescent electrode	Transporting	• Increase the overlap between the electrodes	76
		• Provide a larger uniform driving force	
Heart-shape electrode	Transporting	• Simplifying the circuit wiring	79
		• Transporting in a single direction	
Improved crescent electrode	Transporting	• Increase the overlap between the electrodes	80
		• Both directions transporting	
Combined electrode	Transporting	• Free switch of the droplet operation direction	81
		• Favorable for the layout of the electrodes	
Stripped electrode	Transporting	• Improve droplet transmission efficiency	82
	Splitting	• Parallel droplet production	84
Virtual electrode	Transporting	• The shape of the electrode can be changed	87
		• Meet multiple experimental requirements	
TCC reservoir electrode	Dispensing	• High volume precision and accuracy	88
L-junction electrode	Dispensing	• High volume precision and accuracy	89
		• Fast droplet dispensing: 55–110 ms	
Y-junction electrode	Splitting	• Quick droplet splitting: 35–70 ms	89
		• Operation in a single direction	
Dumbbell electrode	Dispensing	• Improve the consistency of the droplet volume	91
Optimized dispensing electrodes	Dispensing	• High volume precision and accuracy	92
		• Complex operation	
Optimized splitting electrodes	Splitting	• High volume precision and accuracy	92
		• An operation can be done in both directions	

The square electrode is easy to lay out, but there are problems, such as often causing the droplet to be stranded and slowly transported. Based on those problems, Interdigitated and crescent electrodes are proposed to increase the interleaving between electrodes to enhance droplet transport's continuity and speed. Because of the unidirectional transport of the crescent electrode, the improved crescent electrode and the combined electrode realize the transport of the droplets in different directions. For the low accuracy of droplet volume, TCC reservoir, dumbbell electrode, and optimized electrodes provide choices for experiments with requirements of high-accuracy droplet volume. In addition to increasing the speed of droplets, the L-junction and Y-junction electrodes accelerate the dispensing and splitting of droplets while maintaining the consistency and accuracy of the volume of the droplet. In short, the experimental requirements can be met by selecting different shapes of electrodes.

## Conclusions and future outlook

With the accelerated development of DMF and the increasing application fields, new technical requirements are proposed for DMF equipment. Because of the limitations of square electrodes, many electrodes have been designed, becoming an effective means to improve the performance of droplet control. Researchers constantly improve traditional electrodes in current research experiments and applications to meet different needs. Designing driving electrodes of different shapes improved the speed and precision of droplet operation.

Using electrodes for droplet splitting can produce droplets with high volume precision, reducing the errors caused by volume and improving the sample volume accuracy in subsequent experiments. Certain electrodes enable parallel operation of the droplet on the DMF chip, reducing the time to repeat steps and enabling the simultaneous analysis of multiple substances. Decrease the number of electrode control lines to reduce the volume of DMF, which helps achieve the DMF platform's portability. Developing of electrodes with different shapes improves the accuracy and reliability of controlling droplets and accelerates the application of the DMF platform. However, the rule still needs to be clarified due to the lack of universal models or formulas for interpreting different driving electrodes.

Different shapes of electrodes provide researchers with diverse options to conduct experiments better, but there are also some limitations. For example, the special shape of these electrodes arranges less convenient than conventional square electrodes because the symmetry and simple geometry of square electrodes make it possible to achieve the regular arrangement of electrodes of different sizes and achieve droplet manipulation in all directions. In addition, the special-shaped electrodes are not universal and may only be suitable for a single droplet operation. Moreover, due to the relative complexity of the chip fabrication, there is a problem of insufficient precision in chip fabrication in printed circuit technology, which is not able to do for complex shapes or smaller shapes of electrodes. Due to the relative complexity of chip fabrication. The above problems lead to a serious limitation of the application of these electrodes.

The design and selection of electrodes are mostly based on the experiment, and the selections and arrangement are according to the specific requirements. Currently, electrodes with interdigitated edges are popular because they enhance the drive performance and facilitate the arrangement of electrodes on the chip. The experimental requirements are increasing with the application of DMF in many high-precision fields. The electrode design needs to pay more attention to the precision of droplet control, especially the precise control of tiny volume droplets, which is expected to better complete the experiment. In the future, with the development of DMF in the field of portable devices and point-of-care testing, the research will also focus on developing more versatile electrodes suitable for a variety of operations in order to adapt to different operations and various samples.

Droplet control accuracy is improving with the development of large-scale production technology and general DMF instruments. DMF's compatibility, integration, and automation level is increasing, producing a batch of emerging commercial products in biomedical and other aspects.^93^ For example, the DMF-based DNBelab D series of instruments released by MGI are combined with Clustered Regularly Interspaced Short Palindromic Repeats (CRISPR) molecular diagnostic technology to detect COVID-19. Only experimental samples and kits are added manually, and other processes are automatically completed in a closed experimental environment. It can avoid the external environment's interference and completes the process from sample to report automatically and quickly, significantly improving the detection speed. Virus Hunter by Digifluidic Biotech Ltd. includes DMF chips and rapid detection devices. The detection operation can be completed automatically through preset procedures, providing testing services for infectious diseases, health indicators, animal and plant diseases, *etc.* When testing for COVID-19, the experimental process can be completed within 30 minutes.

To pursue miniaturization, high throughput, high integration, high automation and portability is the trend of DMF development in the future. More critically, DMF has characteristics of the open and inclusive. With more and more new technology breakthroughs, DMF will integrate new technologies to improve the performance of DMF and expand its application scope, which is bound to affect multiple fields and has a good prospect.

## Author contributions

Conceptualization, X. W. and Y. T.; writing-original draft preparation, X. W., D. T., L. L. and Z. J.; writing-review and editing, X. W., Q. H. and Y. T.; funding acquisition, validation and supervision, Y. T. All authors have read and agreed to the published version of the manuscript.

## Conflicts of interest

There are no conflicts to declare.

## Supplementary Material
